# Assessment tools for cognitive performance in Parkinson’s disease and its genetic contributors

**DOI:** 10.3389/fneur.2024.1413187

**Published:** 2024-06-26

**Authors:** Ling-Xiao Cao, Wee Lee Kong, Piu Chan, Wei Zhang, Margaret J. Morris, Yue Huang

**Affiliations:** ^1^China National Clinical Research Center for Neurological Diseases, Beijing Tiantan Hospital, Capital Medical University, Beijing, China; ^2^Department of Neurology, Beijing Tiantan Hospital, Capital Medical University, Beijing, China; ^3^Pharmacology Department, School of Biomedical Sciences, Faculty of Medicine and Health, UNSW Sydney, Sydney, NSW, Australia; ^4^Department of Neurobiology, Neurology and Geriatrics, National Clinical Research Center for Geriatric Disorders, Xuanwu Hospital of Capital Medical University, Beijing, China

**Keywords:** Parkinson’s disease, cognitive assessment scale, cognitive decline, genetic association, single nucleotide polymorphism

## Abstract

**Background:**

We have shown that genetic factors associating with motor progression of Parkinson’s disease (PD), but their roles in cognitive function is poorly understood. One reason is that while cognitive performance in PD can be evaluated by various cognitive scales, there is no definitive guide indicating which tool performs better.

**Methods:**

Data were obtained from the Parkinson’s Progression Markers Initiative, where cognitive performance was assessed using five cognitive screening tools, including Symbol Digit Modalities Test (SDMT), Montreal Cognitive Assessment, Benton Judgment of Line Orientation, Modified Semantic Fluency Test, and Letter Number Sequencing Test, at baseline and subsequent annual follow-up visit for 5 years. Genetic data including *ApoE* and other PD risk genetic information were also obtained. We used SPSS-receiver operating characteristic and ANOVA repeated measures to evaluate which cognitive assessment is the best reflecting cognitive performance in PD at early stage and over time. Logistic regression analyses were used to determine the genetic associations with the rapidity of cognitive decline in PD.

**Results:**

SDMT performed better in detecting mild cognitive impairment at baseline (AUC = 0.763), and SDMT was the only tool showing a steady cognitive decline during longitudinal observation. Multigenetic factors significantly associated with cognitive impairment at early stage of the disease (AUC = 0.950) with *IP6K2* rs12497850 more evident, and a significantly faster decline (AUC = 0.831) within 5 years after motor onset, particularly in those carrying *FGF20* rs591323.

**Conclusion:**

SDMT is a preferable cognitive assessment tool for PD and genetic factors synergistically contribute to the cognitive dysfunction in PD.

## 1 Introduction

Parkinson’s disease (PD) is the second most common neurodegenerative disease after Alzheimer’s disease (AD), which affects 1% of people over 65 years and 3% of those over 80 years old (1). Apart from typical motor symptoms including rest tremor, bradykinesia, and rigidity, non-motor symptoms such as cognitive impairment, autonomic dysfunction, hyposmia, and rapid eye movement sleep behavior disorder (RBD) are also important clinical features of PD. Cognitive impairment is one of the most common non-motor symptoms, and its incidence in patients with PD is 2.5 to 6 times higher than that of healthy controls (2). Cognitive functions in PD are heterogeneous, with a subset of patients experiencing reversible cognitive alterations. However, when considering the entire PD patient population, there is a discernible longitudinal decline in cognitive function over time (3–7). The prevalence of mild cognitive impairment (MCI) accounts for up to 40% patients with PD (8), and dementia is observed in 24%–31% of patients with PD (9). Furthermore, more than 10 years following after the diagnosis of PD, over 75% of patients will suffer from dementia (10). Undeniably, cognitive impairment in PD patients drastically reduces their quality of life, imposes significant economic burdens on families and society, making it a pressing and unmet challenge.

Cognitive impairment in PD can be reported by patients and caregivers, or observed by clinicians. However, due to different observation perspectives, consensus on a patient’s cognitive status is often difficult to reach. Therefore, standardized cognitive rating scales are often used in clinical and research settings to assess cognitive function of patients with PD. Currently, there are multiple cognitive assessment scales used in different centers around the world for cognitive screening in PD, including Mini-Mental State Examination (MMSE), Montreal Cognitive Assessment (MoCA), Symbol Digit Modalities Test (SDMT), Benton Judgment of Line Orientation (JLO), Modified Semantic Fluency Test (SF), Letter Number Sequencing Test (LNS), and Addenbrooke’s Cognitive Examination (ACE), etc. (11–15). However, the use of cognitive assessment scales was highly variable between the different studies (16). In addition, to the best of our knowledge, there is no study comparing multiple cognitive assessment scales to evaluate their applicability and accuracy in detecting cognitive impairment in PD. Most studies have compared two cognitive assessment scales in cross-sectional PD cohorts, rather than in a longitudinal fashion. Reliable cognitive assessment is essential to evaluate the progression of cognitive changes in PD and implement timely cognitive intervention so as to deploy staff resources efficiently and improve outcomes. Therefore, it is important to identify the most reliable cognitive assessment tool that reflects cognitive dysfunction in PD patients, which requires the application of multiple cognitive assessment tools to the same longitudinal observation cohort.

Genetic factors have been shown to contribute to the rapidity of motor deterioration in PD (17–20), but the extent of genetic contribution to the cognitive decline in PD is poorly understood, partially due to the lack of a stable cognitive assessment tool for PD. Since the application of genome-wide association studies (GWAS) in PD, more than 90 independent single nucleotide polymorphisms (SNPs) have been identified in association with PD (21, 22), implicating multiple molecular pathways involved in the process of disease initiation. The progressive cognitive decline of PD is likely due to multiple molecular processes, among which *apolipoprotein E* (*ApoE*) has been shown as a vital gene for cognitive performance in PD (23, 24). Other genes such as glucocerebrosidase (*GBA*), microtubule-associated protein tau (*MAPT*), and catechol O-methyltransferase (*COMT*), may also be associated with cognitive decline in PD (25–29). Genes influence cognitive function through diverse pathways. For instance, GBA mutations promote the accumulation and aggregation of α-synuclein, ApoE significantly enhances the accumulation and subsequent deposition of amyloid-β in brain, and COMT is a regulator of synaptic dopamine (23, 27, 30, 31). While SNPs associated with PD may not solely determine the risk of disease onset, they could potentially shape the cognitive patterns exhibited by PD patients. Nonetheless, the intricate relationships between numerous PD related SNPs and cognitive function markers remain understudied and deserve further investigation. Identification of genetic contributions to cognitive deterioration in PD will enable us to better predict cognitive outcomes and eventually offer personalized treatment and care for patients. For this purpose, this study aims to determine a more suitable cognitive assessment scale in a longitudinal PD cohort, and further explore any genetic associations with the rapidity of cognitive decline.

## 2 Materials and methods

### 2.1 Participants

The data containing information of all participants were obtained from Parkinson’s Progression Markers Initiative (PPMI) funded by Michael J. Fox Foundation (MJFF) (32). PPMI was launched in 2011 to enroll participants, and the data pertaining to this study were retrieved from PPMI in January 2022. This study was approved by the scientific committee of PPMI and the Ethics Board of the Beijing Tiantan Hospital, Capital Medical University of China (KY 2018-031-02). The inclusion criteria for PD were as follow: (1) participants were Caucasians; (2) participants were diagnosed with idiopathic PD without other neurological diseases; (3) participants were not diagnosed with prodromal status, or without dopaminergic deficit (SWEDD), as scanned by dopamine transporter deficit on ^123^I ioflupane imaging (DaTscan); (4) participants were evaluated at least five times with missing data representing ≤20%, including baseline evaluation. The inclusion criteria for healthy controls (HCs) were as follows: (1) participants were diagnosed without PD or other neurological diseases; (2) participants had no family history of PD; (3) participants were evaluated at least five times with missing data representing ≤20%, including baseline evaluation. After screening, a total of 306 patients with PD and 99 HCs were included in this study.

### 2.2 Baseline and 5-year follow-up evaluation

#### 2.2.1 Clinical data

##### 2.2.1.1 Baseline evaluation

Unified Parkinson’s Disease Rating Scale (UPDRS) part III (UPDRS-III) was used to assess motor symptoms. MoCA, SDMT, JLO, SF, and LNS were applied to evaluate cognitive function. MoCA is a comprehensive cognitive screening tool that encompasses various aspects including orientation, attention, language, visuospatial, memory, and executive domains. SDMT is a neuropsychological test specifically designed to evaluate attention and processing speed, encompassing diverse domains like attention, visuospatial, memory, and executive function. LNS is tailored to assess working memory and executive function (33), while SF is related to language function. Furthermore, JLO has been applied in PD for examining visuospatial function (34). According to the MCI criteria in PPMI, MCI was delineated as scores on two or more cognitive screening scales, encompassing Hopkins Verbal Learning Test (HVLT), JLO, LNS, SF, and SDMT, that fell more than 1.5 standard deviations below the normal range, while exhibiting no functional impairment stemming from cognitive decline (32). Additionally, this definition aligns with MDS Level 1 criteria (35). For other non-motor symptoms, Rapid Eye Movement Behavior Disorder Screening Questionnaire (RBDSQ), Scales for Outcomes in Parkinson’s Disease-Autonomic (SCOPA-AUT), and Geriatric Depression Scale (GDS) were used for the evaluation of RBD, autonomic dysfunction, and depression, respectively.

##### 2.2.1.2 Five-year follow-up evaluation

Participants from PPMI received regular follow-up evaluations since enrollment at baseline. According to the PPMI protocol, standard follow-up visits were scheduled for every 3 months during the initial year, every 6 months for the subsequent 4 years, and annually thereafter. Although cognitive function was assessed using different scales by different researchers from different PPMI locations, all researchers underwent and completed cognitive assessments training and quality control had been strictly monitored according to standardized protocols, thereby mitigating potential biases. Data on MoCA, SDMT, SF, and LNS scores for cognitive assessment and UPDRS-III for motor assessment were collected at baseline and annually throughout the following 5 years. We identified rapid cognitive and motor declines in patients who experienced changes in scores greater than the 75th percentile of all participants.

#### 2.2.2 Genetic data

Genotyping results for *ApoE* and other PD-associated SNPs were obtained from NeuroX array. NeuroX array is an Illumina Infinium iSelect PD Custom Genotyping array designed for neurological disease studies. Genetic data were processed and analyzed using PLINK software (version 1.9). Quality controls were conducted for all samples and genotypes, and the threshold call rate was 95%. Quality controls of sample processing were determined by comparing the subject’s sex reported by Coriell Institute for Medical Research with the genotypic sex estimated from X chromosome heterogeneity. X chromosome heterogeneity calculations were based on common SNPs from the HapMap Project with <5% genotype deletions and Hardy–Weinberg equilibrium (HWE) *p* values >1 × 10^−6^. Samples containing discrepancies between reported sex and genotypic estimated sex were excluded. To exclude outliers, we performed principal component analysis (PCA) based on genotype using GCTA (version 1.93). Finally, we obtained genotype information of *ApoE* and 44 PD-risk SNPs from NeuroX array database of PPMI (Supplementary Table S1).

### 2.3 Statistical analysis

IBM SPSS 26 was used for statistical analysis in this study. We standardized the cognitive testing scores by z transformation using mean and standard deviation for PD and HCs, and z scores were calculated. Chi-squared test and one-way ANOVA were used for comparative analysis between groups. Receiver operating characteristic (ROC) curve analysis was used to evaluate the diagnostic value for MCI. Disease duration, gender, years of education, and age at onset were selected as covariates. The test–retest reliability was measured by intraclass correlation coefficients (ICC). Data between baseline scores and scores at each follow-up visit over 5 years were analyzed using ANOVA repeated measures, and Bonferroni’s correction was used for *post hoc* analysis.

## 3 Results

### 3.1 Baseline data observation

A total of 306 PD patients (mean age 61.0 ± 9.5 years; 61.8% male) and 99 HCs (mean age 60.4 ± 11.1 years; 59.6% male) were included in this study (Supplementary Table S2). In the PD cohort, the mean SDMT, MoCA, LNS, SF, and JLO scores were 41.4 ± 10.0, 26.7 ± 2.6, 10.5 ± 2.8, 22.0 ± 5.4, and 12.7 ± 2.4, respectively, and the mean UPDRS-III score was 19.6 ± 8.8 at baseline (Supplementary Table S2). SDMT and MoCA scores in PD patients were significantly lower than those of HCs (*p* < 0.001), while there were no significant differences between PD and HCs in LNS, SF, and JLO scores (Supplementary Table S2). As for motor and other non-motor variables, apart from GDS, there were significant differences between PD and healthy controls in UPDRS-III, RBDSQ, and SCOPA-AUT scores (*p* < 0.001). Among the patients with PD, 35 participants (11.4%) were classified as MCI. SDMT showed better performance in detecting MCI than the other four scales with the highest area under curve (AUC = 0.763; Supplementary Figure S1; Supplementary Table S3). Among the 306 PD patients, genotype data were available on 197 patients.

### 3.2 Longitudinal cognitive observation

**Figure 1:**
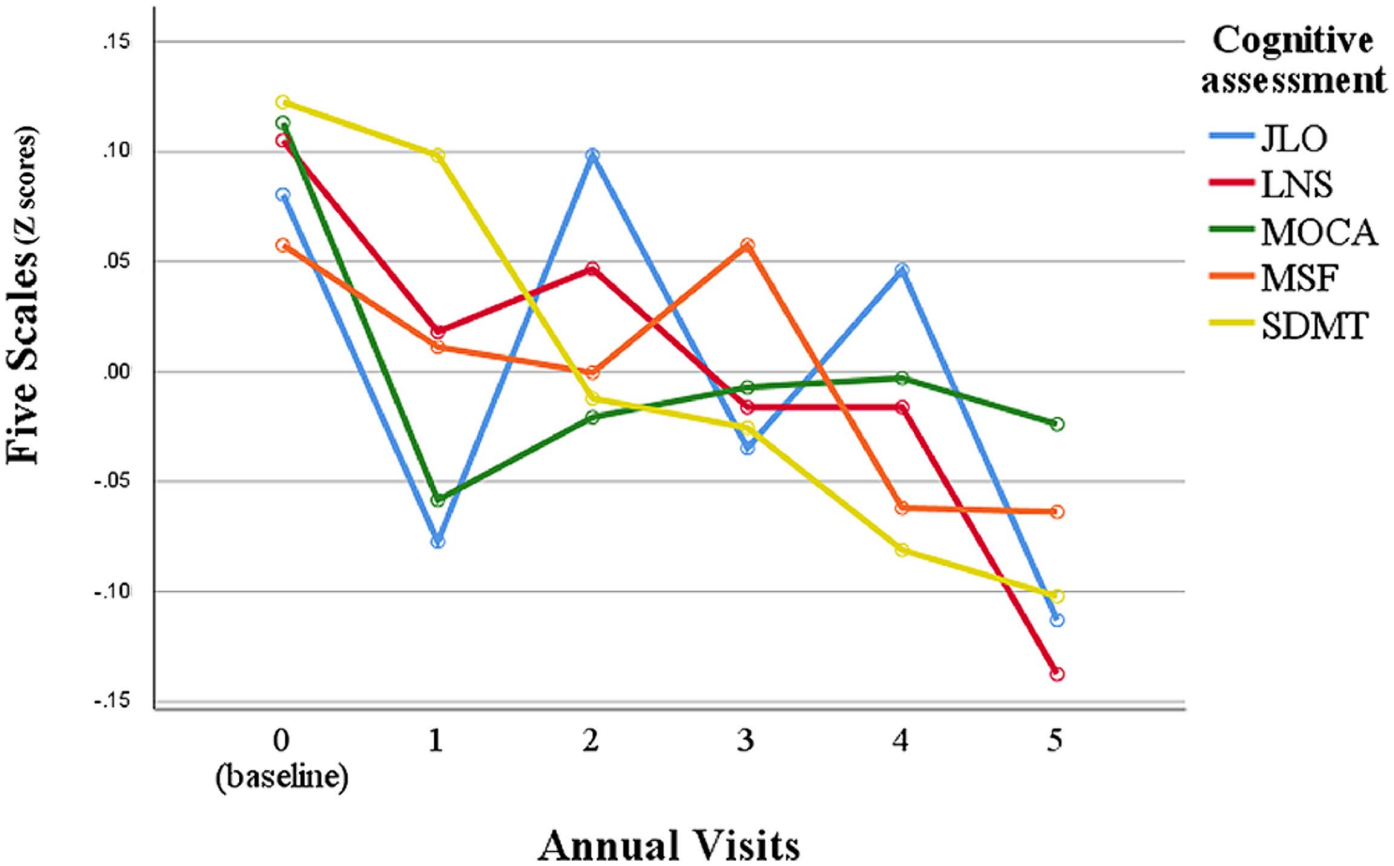
Longitudinal observation of the performance of each cognitive assessment scale in PD patients. Z scores were calculated by z transformation using mean and standard deviation. JLO, Benton Judgment of Line Orientation; MoCA, Montreal Cognitive Assessment; SDMT, Symbol Digit Modalities Test; LNS, Letter Number Sequencing Test; SF, Modified Semantic Fluency Test.

Analyses revealed that SDMT, LNS, and JLO scores in PD patients significantly declined over 5 years (*p* < 0.001; Table 1; Figure 1). Entire PD patient population shows a discernible longitudinal decline in cognitive function (5–7, 36). In this study, SDMT scores showed a steady decrease over time, while the other four scales had more fluctuating declines over 5 years of follow-up (Figure 1). JLO scores had significant changes at year one, three, and five follow-ups compared to the previous visit, and overall, the scores were unstable (Figure 1). MoCA scores decreased at year one follow-up and remained steady thereafter (Figure 1). LNS scores showed a step-wise decline with plateaus at year one to year two and year three to year four (Figure 1). Repeated ANOVA analysis also showed that SDMT was the more suitable cognitive scale to assess cognitive function in PD (Figure 1). In addition, SDMT had a better reliability based on intraclass correlation coefficients (ICC = 0.75), while ICC for MoCA, JLO, LNS, and SF were 0.68, 0.58, 0.69, and 0.65, respectively. Additionally, only SDMT was significantly correlated with rapid motor progression (*p* < 0.010; Supplementary Table S4). In the healthy control group, only MoCA score showed significant changes over 5 years (*p* < 0.001; Table 1), while SDMT, LNS, JLO, and SF scores did not change significantly during the 5-year follow-up.

**Table 1:** Cognitive performance at baseline and over 5 years in PD and HC.

Cognitive assessments	Visits						*P*-value^*^
	Baseline	Year 1	Year 2	Year 3	Year 4	Year 5	
**PD**							
SDMT score, mean (SD)	41.35 (9.98)	41.08 (10.05)	39.84 (10.48)	39.69 (11.80)	39.07 (11.85)	38.83 (12.52)	**<0.001**
MoCA score, mean (SD)	26.69 (2.64)	26.15 (3.03)	26.27 (2.97)	26.31 (3.03)	26.31 (3.32)	26.26 (3.65)	0.010
LNS score, mean (SD)	10.50 (2.80)	10.25 (2.64)	10.33 (2.78)	10.15 (2.86)	10.15 (2.98)	9.80 (3.04)	**<0.001**
SF score, mean (SD)	22.02 (5.35)	21.76 (5.65)	22.70 (5.70)	22.02 (5.35)	21.36 (5.68)	21.35 (5.72)	0.049
JLO score, mean (SD)	12.68 (2.40)	12.31 (2.37)	12.73 (2.24)	12.41 (2.37)	12.60 (2.47)	12.20 (2.47)	**<0.001**
**HC**							
SDMT score, mean (SD)	47.24 (10.98)	48.55 (10.71)	46.49 (10.39)	47.95 (11.30)	47.29 (11.17)	47.08 (10.89)	0.120
MoCA score, mean (SD)	28.09 (1.02)	27.15 (2.03)	27.15 (2.21)	27.34 (2.18)	27.55 (2.25)	27.49 (2.07)	**<0.001**
LNS score, mean (SD)	10.93 (2.49)	11.12 (2.73)	11.14 (2.56)	11.28 (2.73)	11.15 (2.68)	11.11 (2.92)	0.713
SF score, mean (SD)	21.10 (5.44)	21.70 (5.47)	21.68 (6.79)	21.10 (5.44)	21.35 (5.64)	21.88 (6.40)	0.424
JLO score, mean (SD)	13.12 (2.09)	12.60 (2.40)	12.93 (2.51)	12.76 (2.14)	13.05 (2.55)	12.77 (2.26)	0.182

All data are shown as mean ± standard deviation. ^*^p-values were calculated using ANOVA repeated measures, *p* < 0.01 was taken as cutoff value for significance after multiple correction. PD, Parkinson’s disease; HC, healthy control; JLO, Benton Judgment of Line Orientation; MoCA, Montreal Cognitive Assessment; SDMT, Symbol Digit Modalities Test; LNS, Letter Number Sequencing Test; SF, Modified Semantic Fluency Test; SD, Standard Deviation. Significant *p* values were emphasized in bold.

**Figure 2:**
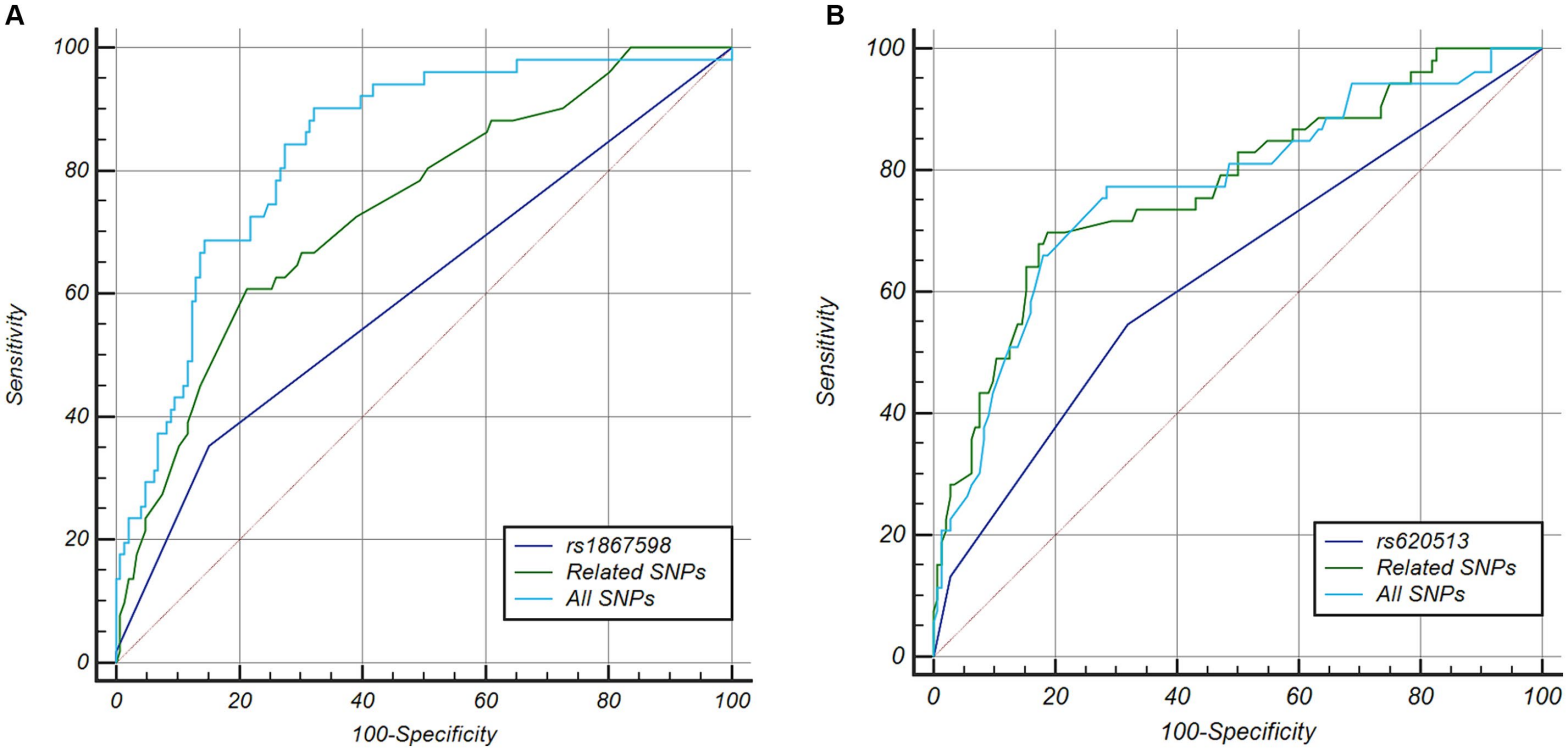
ROC curve analysis of five cognitive scales and genetic factors for detecting and predicting cognitive function in PD. **(A)** For predicting cognitive impairment at motor onset of PD, the AUC of rs12497850 was 0.661, four related SNPs reach a higher AUC at 0.809, but less than all SNPs together (AUC = 0.950). **(B)** For predicting cognitive impairment within 5 years of PD diagnosis, the AUC of rs591323 is 0.604, seven related SNPs reached a higher AUC at 0.723, but less than all SNPs together (AUC = 0.831). PD, Parkinson’s disease; JLO, Benton Judgment of Line Orientation; MoCA, Montreal Cognitive Assessment; SDMT, Symbol Digit Modalities Test; LNS, Letter Number Sequencing Test; SF, Modified Semantic Fluency Test; ROC, receiver operating characteristic; AUC, area under curve.

After determining that SDMT was the most suitable measure reflecting cognitive function in patients with PD among five cognitive screening scores at the early stage of the disease, we identified rapid cognitive decline in those patients who experienced changes in scores greater than the 75th percentile of all participants. There were no significant differences in demographics and cognitive function at baseline between the rapid cognitive decline group and others (Supplementary Table S5). Patients with rapid cognitive decline in SDMT also showed significant declines in MoCA and JLO. Rapid cognitive progressors had higher RBDSQ and SCOPA-AUT scores, and they also had faster motor symptom decline in UPDRS-III. We then investigated genetic factors contributing to cognitive performance. Four SNPs (rs4653767, rs6808178, rs12497850, and rs14235) were associated with poorer cognitive performance at baseline at *p* < 0.05, and only rs12497850 (residing in *IP6K2*, *p* = 0.0009; Table 2) was significantly associated with weaker cognitive performance after correction for multiple testing (*p* < 0.0011). For cognitive deterioration over the disease course, six SNPs (rs115185635, rs11724635, rs591323, rs13294100, rs2251776, and rs12456492) and *ApoE* ε4 were associated with faster cognitive decline at *p* < 0.05, and only rs591323 (residing in *FGF20*, *p* = 0.0007; Table 2) passed correction for multiple testing (*p* < 0.0011). *IP6K2* rs12497850 yielded a model with an AUC of 0.661 for predicting cognitive impairment at baseline, while related SNPs reached a higher AUC at 0.809, and all 44 SNPs with *ApoE* reached a better prediction with AUC at 0.950 (Figure 2A). For the prediction of cognitive decline after disease onset, the AUC for rs591323 was 0.604, it reached 0.732 when considering related SNPs, and AUC for all 44 SNPs with *ApoE* was 0.831 (Figure 2B).

**Table 2:** Genetic associations with cognitive performance at baseline and rapid cognitive decline in PD.

	SNP	Related gene	Beta	OR	*P*-value^*^
Cognitive performance at baseline	rs4653767	*ITPKB*	0.854	2.350 (1.146–4.818)	0.020
	rs6808178	*LINC00693*	0.826	2.285 (1.160–4.502)	0.017
	rs12497850	*IP6K2*	1.283	3.609 (1.697–7.675)	**0.0009**
	rs14235	*BCKDK/STX1B*	−0.741	0.477 (0.236–0.963)	0.039
	ε4	*ApoE*	1.978	7.226	0.084
Rapid cognitive decline	rs115185635	*CHMP2B*	−2.348	0.096 (0.013–0.681)	0.019
	rs11724635	*BST1*	0.599	1.820 (1.124–2.947)	0.015
	rs591323	*FGF20*	1.046	2.847 (1.552–5.222)	**0.0007**
	rs13294100	*SH3GL2*	0.543	1.721 (1.010–2.933)	0.046
	rs2251776	*MIPOL1*	0.717	2.048 (1.235–3.396)	0.005
	rs12456492	*RIT2*	0.629	1.876 (1.109–3.172)	0.019
	ε4	*ApoE*	0.900	2.459 (1.188–5.090)	0.015

^*^p-values were calculated using binary logistic regression, *p* < 0.0011 was taken as cutoff value for significance after multiple correction. PD, Parkinson’s disease; SNP, single nucleotide polymorphism; OR, odds ratio. Significant *p* values were emphasized in bold.

## 4 Discussion

In this study, we found that the SDMT may serve as a more suitable measure for reflecting cognitive function in patients PD. This is due to its steady downward trend, superior reliability, enhanced capability in detecting MCI, and significant association with motor progression. *IP6K2* was associated with cognitive impairment prior to the onset of motor symptoms and *FGF20* was associated with rapid cognitive decline after the onset of motor symptoms in PD.

There is a wide range of severity, progression rate, and affected cognitive domains in PD, from subjective cognitive complaints without objective evidence, MCI and dementia (37). The molecular basis of cognitive impairment in PD is likely related to neurotransmitter systems such as dopamine, acetylcholine, norepinephrine, and serotonin. Among them, dopamine depletion may lead to deficiencies in attention, working memory, planning, and response inhibition, while cholinergic loss has been associated with the decline of memory, language, and visuospatial function (38). Eventually, multiple domains were reported to be impaired in PD with cognitive impairment, especially executive function, followed by memory, attention, and visuospatial functions (39, 40). In addition, early cognitive function changes in PD primarily manifest in executive and visuospatial functions (31).

We found that SDMT was a more suitable tool not only for initial cognitive screening, but also for tracking cognitive changes over the course of PD. SDMT is a digit substitution test that requires the participant to swiftly pair each symbol with its corresponding number, adhering to the matching pattern provided at the top of the page. It is a neuropsychological test that assesses attention and processing speed, which involves multiple domains of attention, visuospatial, memory, and executive function (41). The mean change in SDMT from baseline to 5-year follow-up was 2.1 in a previous study (42), which was consistent to our findings (2.5). In this study, we found that MoCA scores declined most obviously at the first-year visit, and then remained stable in the following years, while it was a sensitive cognitive assessment tool in controls (Table 1), suggesting a ceiling effect of cognitive assessment in PD. MoCA, as a cognition screening instrument, was devised based on clinical insights into the typical impairment domains encountered in MCI, ensuring its optimal suitability for screening purposes. This comprehensive cognitive screening tool encompasses various cognitive domains such as orientation, attention, language, visuospatial skills, memory, and executive functions. MoCA has exhibited good test–retest reliability, internal consistency, and equivalence (43). It has good interrater reliability (0.81) and test–retest reliability (0.79) in PD (44). A 30-month follow-up PD study showed that MoCA scores were significantly lower compared with those at baseline (45). However, some MoCA subtests may be so challenging for PD patients that there may be floor effects (46, 47). Furthermore, executive and visuospatial domains tend to be more vulnerable during the early stages of the disease, which may result in potentially less sensitivity in detecting declines in the total scores of the MoCA compared to SDMT (31). As the disease advances to later stages, a broader range of cognitive domains are impacted, and MoCA may demonstrate better performance, but it needs further validation. LNS is influenced by various factors, including reading level, digit span forward and backward, arithmetic, and visual spatial learning. It serves as a scale tailored specifically for assessing processing speed and visual spatial working memory (33, 48). SF necessitates the proficiency in accessing and retrieving semantic knowledge, and it maintains a profound association with semantic memory and language function (49). Both LNS and SF have been used in detecting cognitive function in PD (25, 50, 51). Compared to SDMT, SF and LNS scores decreased significantly later in the fourth and fifth-year follow-up. Thus, LNS and SF seem to be more suitable for tracking related cognitive domains in PD for long-term follow-up. Moreover, JLO is regarded as a reliable assessment tool for evaluating visuospatial perception, extensively utilized in the context of PD to examine visuospatial function (34). Although JLO scores decreased significantly across 5 years, they fluctuated during follow-ups, which made JLO less stable for long-term follow-up evaluations. Despite the common use of MMSE, MMSE in not included in PPMI database perhaps due to its copyright, and it is less recommended in PD because of its multiple shortcomings, including insufficient executive and visuospatial components and less sensitivity in capturing mild degrees of deterioration (52).

Interestingly, SDMT appeared to be more suitable for PD cognitive assessment, although MoCA is currently more widely used than SDMT. SDMT has been reported as a stable screening scale in the same PPMI cohort in several studies (53, 54). It has been convincingly demonstrated that SDMT outperforms other scales in conducting longitudinal observations for another movement disorder, specifically Huntington’s disease (55, 56). Furthermore, SDMT can improve the sensitivity to detect vascular cognitive impairment (VCI) compared to MMSE and MoCA (57), and performing SDMT consumes far less time than MoCA and MMSE. In this study, SDMT exhibited superior reliability, enhanced performance in detecting MCI, and a significant association with motor progression. Therefore, we propose that SDMT may be more suitable to evaluate cognitive function in PD compared to MoCA, JLO, LNS, and SF. The reason may be that the cognitive domains detected by SDMT are more substantially and gradually affected by the process of PD, which makes SDMT a better cognitive assessment tool in both cross-sectional screening and longitudinal follow-up tracking, especially at early stage of the disease (42, 58). However, despite the fact that visuomotor impairment rarely affects SDMT performance, SDMT may still be influenced by bradykinesia (42, 58). To minimize the impact of motor symptoms to the utmost extent, we collect data annually, primarily focusing on evaluating longitudinal changes in cognitive scales rather than cross-sectional performance.

There is increasing evidence suggesting that genetic factors are key drivers of cognitive decline in PD. Variants in *ApoE*, *GBA*, *MAPT*, α-synuclein (*SNCA*) have been proven to be associated with cognitive decline and dementia in PD (23, 25, 26, 59–61). However, the question of whether PD-related SNPs recently discovered by GWAS are associated with cognitive performance warrants attention. In this study, we found that *IP6K2* rs12497850 was associated with cognitive impairment at the time of motor symptoms onset, and *FGF20* rs591323 was associated with more rapid cognitive decline after motor symptoms onset. After analysis in 3DSNP,^1^ rs12497850 may serve as a potential genic enhancer in tissues such as the hippocampus, despite being an intron variant (62). While rs591323 is also an intron variant, the extent to which its variation impacts the activity of FGF20 remains to be investigated (63). In our previous study, *IP6K2* rs12497850 also displayed a potential association with motor progression, yet it did not meet the threshold for significance after multiple comparison correction (17). *IP6K2* has been shown to participate in the process of cell death and apoptosis. It functions in maintaining mitochondrial homeostasis, promoting neuroprotection, and regulating Purkinje cell morphology (64–66). Experimental models have demonstrated that Purkinje cell dysfunction may be associated with cognitive function in the early stage of disease (67). With the accumulation of pathological protein(s), this effect might reach plateau and become less obvious, as *IP6K2* is not associated with cognitive deterioration after motor onset of PD. *FGF20* encodes a protein of the fibroblast growth factor family. FGF20 is preferentially expressed in the substantia nigra pars compacta of the brain and significantly improves the survival of midbrain dopaminergic neurons (68). *FGF20* mutations are also related to decreases in hippocampal volume and diminished verbal episodic memory (69), which could account for our findings. Some studies have shown that *ApoE* ε4 carriers in PD had a faster cognitive deterioration rate (23, 25, 26, 60, 70). *ApoE* ε4 has been shown to accumulate along with the deposition of β amyloid, and it promotes α-synuclein aggregation, thus being associated with the severity of Lewy body pathology (71, 72), which may be the molecular mechanisms of worsening cognitive function in *ApoE* ε4 carriers of PD patients. In this study, *ApoE* was associated with rapid cognitive decline in PD with *p* < 0.05, but it failed to pass multiple testing correction. The lack of *ApoE* association with cognitive deficits in PD in another study was attributed to the early disease stage of that cohort (59). As participants in PPMI are mostly at an early stage of PD, associations between *ApoE* and cognitive changes may become obvious with longer observation windows.

There are several limitations in this study. First, although we have compared five different cognitive scales to identify an optimal method for cognitive assessment, we did not include other cognitive assessments, such as ACE-III (73), etc., because they were not included in the PPMI protocol. Therefore, we cannot comment on whether the use of SDMT has an advantage over ACE-III in cognitive evaluation in patients with PD. Second, patients enrolled in this study were mostly at early stage of PD, and the follow-up period was only up to 5 years. Cognitive changes would become more obvious in the middle and late stages of PD, and different molecular mechanisms might be involved at different disease stages. Third, genetic information was not available for the entire PPMI cohort, and other ethnicities were not considered in this study. Finally, the number of participants in this study cohort is relatively small. Therefore, further studies should incorporate a longer follow-up period, a larger multi-ethnic sample size, and a broader range of cognitive screening scales.

## 5 Conclusion

Our study indicates that SDMT is a preferable cognitive screening tool for tracking cognitive function in both cross-sectional and longitudinal studies of patients with PD during the early stages of the disease. Furthermore, the rapidity of cognitive decline in PD could be attributable to heterogeneous genetic factors, which may need to be verified in other PD cohorts with comprehensive clinical and genetic information.

## Data availability statement

The original contributions presented in the study are included in the article/Supplementary material, further inquiries can be directed to the corresponding author.

## Ethics statement

The studies involving humans were approved by Beijing Tiantan Hospital and PPMI. The studies were conducted in accordance with the local legislation and institutional requirements. The participants provided their written informed consent to participate in this study.

## Author contributions

L-XC: Formal analysis, Investigation, Methodology, Validation, Writing – original draft. WK: Investigation, Validation, Writing – original draft. PC: Funding acquisition, Methodology, Supervision, Validation, Writing – review & editing. WZ: Resources, Supervision, Validation, Writing – review & editing. MM: Resources, Supervision, Writing – review & editing. YH: Conceptualization, Funding acquisition, Project administration, Resources, Supervision, Writing – review & editing.

## Footnotes

^1^ https://omic.tech/
