# Supplementary material for: Assessment tools for cognitive performance in Parkinson’s disease and its genetic contributors
Source: Front Neurol. 2024 Jun 26;15:1413187. doi: 10.3389/fneur.2024.1413187 (PMC11233456; doi:10.3389/fneur.2024.1413187)
Supplement: Supplementary file 1 [file Table_1.DOCX]

Supplementary Material

# Supplementary Figure


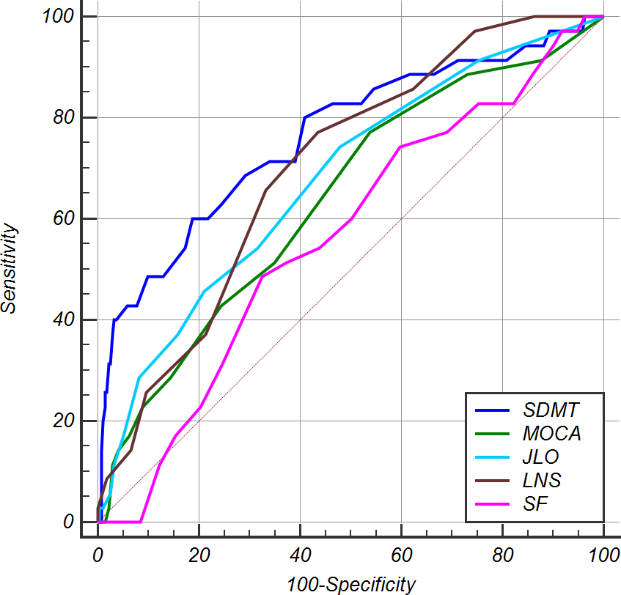


**Supplementary Figure 1. ROC curve analysis of five cognitive scales for detecting MCI in PD**

ROC curve analysis shows SDMT exhibits a better performance in detecting MCI (AUC=0.763) compared with other four scales. Abbreviations: MCI, mild cognitive impairment; PD, Parkinson’s disease; JLO, Benton Judgement of Line Orientation; MoCA, Montreal Cognitive Assessment; SDMT, Symbol Digit Modalities Test; LNS, Letter Number Sequencing Test; SF, Modified Semantic Fluency Test; ROC, receiver operating characteristic; AUC, area under curve.

# Supplementary Tables

**Supplementary Table 1. Summary of Genetic Variants Selected in This Study**

| **No.** | **SNP** | **CHR** | **Position (Bp)** | **EA/AA** | **EA frequency** | **Related Genetic Loci** |
| --- | --- | --- | --- | --- | --- | --- |
| 1 | rs823118 | 1 | 205723572 | C/T | 0.315 | *NUCKS1* |
| 2 | rs10797576 | 1 | 232664611 | T/C | 0.137 | *SIPA1L2* |
| 3 | rs4653767 | 1 | 226916078 | C/T | 0.315 | *ITPKB* |
| 4 | rs6430538 | 2 | 135539967 | T/C | 0.488 | *ACMSD* |
| 5 | rs34043159 | 2 | 102413116 | C/T | 0.352 | *IL1R2* |
| 6 | rs353116 | 2 | 166133632 | T/C | 0.385 | *SCN2A* |
| 7 | rs6808178 | 3 | 28705690 | T/C | 0.378 | *LINC00693* |
| 8 | rs115185635 | 3 | 87520857 | C/G | 0.036 | *CHMP2B* |
| 9 | rs12637471 | 3 | 182762437 | A/G | 0.219 | *MCCC1* |
| 10 | rs4073221 | 3 | 18277488 | G/T | 0.132 | *SATB1* |
| 11 | rs12497850 | 3 | 48748989 | G/T | 0.347 | *IP6K2* |
| 12 | rs34311866 | 4 | 951947 | C/T | 0.199 | *TMEM175* |
| 13 | rs11724635 | 4 | 15737101 | C/A | 0.437 | *BST1* |
| 14 | rs6812193 | 4 | 77198986 | T/C | 0.398 | *FAM47E/SCARB2* |
| 15 | rs356182 | 4 | 90626111 | G/A | 0.375 | *SNCA* |
| 16 | rs78738012 | 4 | 114360372 | C/T | 0.106 | *CAMK2D* |
| 17 | rs11950533 | 5 | 134199105 | A/C | 0.104 | *C5orf24* |
| 18 | rs2694528 | 5 | 60273923 | C/A | 0.115 | *NDUFAF2* |
| 19 | rs12528068 | 6 | 72487762 | T/C | 0.285 | *RIMS1* |
| 20 | rs997368 | 6 | 112243291 | A/G | 0.803 | *FYN* |
| 21 | rs9468199 | 6 | 27681215 | A/G | 0.172 | *ZNF184* |
| 22 | rs199347 | 7 | 23293746 | G/A | 0.368 | *GPNMB* |
| 23 | rs591323 | 8 | 16697091 | A/G | 0.293 | *FGF20* |
| 24 | rs2280104 | 8 | 22525980 | T/C | 0.367 | *SORBS3* |
| 25 | rs13294100 | 9 | 17579690 | T/G | 0.371 | *SH3GL2* |
| 26 | rs10748818 | 10 | 104015279 | A/G | 0.851 | *GBF1* |
| 27 | rs10906923 | 10 | 15569598 | C/A | 0.306 | *FAM171A1* |
| 28 | rs3793947 | 11 | 83544472 | A/G | 0.463 | *DLG2* |
| 29 | rs329648 | 11 | 133765367 | T/C | 0.327 | *MIR4697* |
| 30 | rs11610045 | 12 | 133063768 | A/G | 0.485 | *FBRSL1* |
| 31 | rs76904798 | 12 | 40614434 | T/C | 0.132 | *LRRK2* |
| 32 | rs11060180 | 12 | 123303586 | G/A | 0.45 | *OGFOD2* |
| 33 | rs9568188 | 13 | 49927732 | T/C | 0.747 | *CAB39L* |
| 34 | rs4771268 | 13 | 97865021 | T/C | 0.237 | *MBNL2* |
| 35 | rs12147950 | 14 | 37989270 | T/C | 0·438 | *MIPOL1* |
| 36 | rs11158026 | 14 | 55348869 | T/C | 0.307 | *GCH1* |
| 37 | rs8005172 | 14 | 88472612 | T/C | 0.424 | *GALC* |
| 38 | rs2414739 | 15 | 61994134 | G/A | 0.292 | *VPS13C* |
| 39 | rs14235 | 16 | 31121793 | A/G | 0.397 | *BCKDK/STX1B* |
| 40 | rs11343 | 16 | 19279464 | T/G | 0.454 | *COQ7* |
| 41 | rs4784227 | 16 | 52599188 | T/C | 0.265 | *TOX3* |
| 42 | rs17649553 | 17 | 43994648 | T/C | 0.232 | *MAPT* |
| 43 | rs12456492 | 18 | 40673380 | G/A | 0.332 | *RIT2* |
| 44 | rs2248244 | 21 | 38852361 | G/A | 0.283 | *DYRK1A* |

Location and related gene (s) for 44 variants selected in this study based on GWAS meta-analysis. Abbreviations: SNP, single nucleotide polymorphism; Chr, chromosome; GWAS, genome-wide association studies; EA/AA, Effective allele/alternate allele

**Supplementary Table 2. Demographic and Clinical Characteristics of the Cohorts at Baseline**

| **Clinical Characteristics** | **PD** | **HC** | ***P* value^*^** |
| --- | --- | --- | --- |
| Number of people, No. (%) | 306 | 99 | NA |
| Male, No. (%) | 189 (61.8%) | 59 (59.6%) | NA |
| Age at enrollment, mean (SD) | 61.0 (9.5) | 60.4 (11.1) | 0.651 |
| Disease du ration, month, mean (SD) | 29.2 (25.4) | NA | NA |
| Years of education, mean (SD) | 14.9 (4.3) | 15.6 (3.7) | 0.181 |
| SDMT, mean (SD) | 41.4 (10.0) | 47.2 (11.0) | **<0.001** |
| LNS, mean (SD) | 10.5(2.8) | 11.0 (2.5) | 0.171 |
| SF, mean (SD) | 22.0 (5.4) | 21.1 (5.4) | 0.139 |
| JLO, mean (SD) | 12.7 (2.4) | 13.1 (2.1) | 0.104 |
| MoCA, mean (SD) | 26.7 (2.6) | 28.1 (1.0) | **<0.001** |
| UPDRS-III, mean (SD) | 19.6 (8.8) | 2.1 (1.2) | **<0.001** |
| RBDSQ, mean (SD) | 5.5 (3.0) | 3.7 (2.3) | **<0.001** |
| SCOPA-AUT, mean (SD) | 5.4 (1.6) | 5.2 (1.2) | **<0.001** |
| GDS, mean (SD) | 15.5 (10.1) | 9.9 (7.7) | 0.241 |

**^*^***p* values were calculated using one-way ANOVA analysis. Abbreviations: PD, Parkinson’s disease; HC, Healthy control; SDMT, Symbol Digit Modalities Test; LNS, Letter Number Sequencing Test; SF, Modified Semantic Fluency Test; JLO, Benton Judgement of Line Orientation; MoCA, Montreal Cognitive Assessment; UPDRS-III, Unified Parkinson’s Disease Rating Scale part III; RBDSQ, Rapid Eye Movement Behavior Disorder Screening Questionnaire; SCOPA-AUT, Scales for Outcomes in Parkinson’s disease - Autonomic; GDS, Geriatric Depression Scale; SD, Standard deviation.

| **Cognitive assessment** | **AUC** | **Youden's index** | **Sensitivity**  **(%)** | **Specificity**  **(%)** | **PLR** | **NLR** | **PPV (%)** | **NPV (%)** |
| --- | --- | --- | --- | --- | --- | --- | --- | --- |
| JLO | 0.678 | 0.2632 | 74.29 | 52.03 | 1.55 | 0.49 | 16.7 | 94.0 |
| MoCA | 0.640 | 0.2327 | 77.14 | 46.13 | 1.43 | 0.5 | 15.6 | 94.0 |
| SDMT | 0.763 | 0.4118 | 60.00 | 81.18 | 3.19 | 0.49 | 29.2 | 94.0 |
| LNS | 0.702 | 0.3360 | 77.14 | 56.46 | 1.77 | 0.4 | 18.6 | 95.0 |
| SF | 0.563 | 0.1610 | 48.57 | 67.53 | 1.5 | 0.76 | 16.2 | 91.0 |

**Supplementary Table 3. Predictive vales of different cognitive assessment tools in detecting MCI in PD**

Abbreviations: MCI, mild cognitive impairment; PD, Parkinson’s disease; JLO, Benton Judgement of Line Orientation; MoCA, Montreal Cognitive Assessment; SDMT, Symbol Digit Modalities Test; LNS, Letter Number Sequencing Test; SF, Modified Semantic Fluency Test; AUC, area under curve; PLR, positive likelihood ratio; NLR, negative likelihood ratio; PPV, positive predictive value; NPV, negative predictive value.

| **Cognitive assessment** | **Beta** | **OR** | ***P* value*** |
| --- | --- | --- | --- |
| JLO | 0.152 | 1.164(1.007-1.344) | 0.016 |
| MoCA | -0.016 | 0.984(0.890-1.088) | 0.757 |
| SDMT | 0.057 | 1.059(1.020-1.099) | **<0.001** |
| LNS | 0.043 | 1.043(0.914-1.192) | 0.530 |
| SF | -0.07 | 0.932(0.871-0.997) | 0.023 |

**Supplementary Table 4. Cognitive associations with rapid motor deterioration in PD**

* *p* values were calculated using binary logistic regression, *p*<0.01 after multiple correction. Abbreviations: PD, Parkinson’s disease; JLO, Benton Judgement of Line Orientation; MoCA, Montreal Cognitive Assessment; SDMT, Symbol Digit Modalities Test; LNS, Letter Number Sequencing Test; SF, Modified Semantic Fluency Test; OR, odds ratio

**Supplementary Table 5. Characteristic Clinical Features of PD Between Rapid Cognitive Deteriorators and the Others at Baseline and Changes Overtime**

| **Clinical Characteristics** | **Rapid Deteriorators** | **Others** | ***P* value*** |
| --- | --- | --- | --- |
| ***Cross Sectional Observation at Baseline*** | | | |
| Male, No. (%) | 43(68.3%) | 90 (67.1%) | 0.880 |
| Disease duration, month, mean (SD) | 25.8 (16.9) | 20.4 (17.1) | 0.036 |
| Years of education, mean (SD) | 15.4 (3.9) | 15.3 (3.8) | 0.900 |
| RBDSQ, mean (SD) | 6.2 (3.0) | 5.0 (2.7) | **0.007** |
| SCOPA-AUT, mean (SD) | 16.6 (11.4) | 12.9 (7.8) | **0.009** |
| GDS, mean (SD) | 5.3 (1.5) | 5.1 (1.5) | 0.382 |
| SDMT, mean (SD) | 42.6 (10.7) | 41.9 (8.0) | 0.579 |
| LNS, mean (SD) | 10.4(2.5) | 10.9 (2.7) | 0.204 |
| SF, mean (SD) | 22.0 (5.3) | 21.6 (5.4) | 0.856 |
| JLO, mean (SD) | 13.0 (2.3) | 13.2 (1.8) | 0.580 |
| MoCA, mean (SD) | 26.9 (2.3) | 27.1 (2.3) | 0.655 |
| UPDRS-III, mean (SD) | 20.3 (7.4) | 20.2 (8.5) | 0.971 |
| ***Longitudinal Observation after Five Years*** | | | |
| *SDMT change, mean (SD)* | 11.4 (7.2) | -2.7 (5.5) | **<0.001** |
| *LNS change, mean (SD)* | 0.84 (2.3) | 0.52 (2.5) | 0.395 |
| *SF change, mean (SD)* | 1.32 (5.4) | 0.53 (5.5) | 0.350 |
| *JLO change, mean (SD)* | 1.24 (2.7) | 0.32 (1.7) | **0.005** |
| *MoCA change, mean (SD)* | 1.9 (4.8) | -0.3 (2.6) | **<0.001** |
| *UPDRS-III change, mean (SD)* | 13.4 (15.2) | 6.2 (11.5) | **<0.001** |

**^*^***p* values were calculated using one-way ANOVA analysis. Abbreviations: PD, Parkinson’s disease; SDMT, Symbol Digit Modalities Test; LNS, Letter Number Sequencing Test; SF, Modified Semantic Fluency Test; JLO, Benton Judgement of Line Orientation; MoCA, Montreal Cognitive Assessment; UPDRS-III, Unified Parkinson’s Disease Rating Scale part III; RBDSQ, Rapid Eye Movement Behavior Disorder Screening Questionnaire; SCOPA-AUT, Scales for Outcomes in Parkinson’s disease - Autonomic; GDS, Geriatric Depression Scale; SD, Standard deviation.
